# Antimicrobial Resistant Coagulase-Negative Staphylococci Carried by House Flies (*Musca domestica*) Captured in Swine and Poultry Farms

**DOI:** 10.3390/antibiotics12040636

**Published:** 2023-03-23

**Authors:** Fabrizio Bertelloni, Giulia Cagnoli, Flavio Bresciani, Bruno Scotti, Luca Lazzerini, Marco Marcucci, Giuseppe Colombani, Valentina Virginia Ebani

**Affiliations:** 1 Department of Veterinary Science, University of Pisa, Viale delle Piagge 2, 56124 Pisa, Italy; 2 Sede Sicurezza Alimentare e Sanità Pubblica Veterinaria, Zona Versilia, Azienda Usl Toscana Nord Ovest, Via Martiri di S. Anna 12, 55045 Pietrasanta, Italy; 3 Sede Sicurezza Alimentare e Sanità Pubblica Veterinaria, Zona Valle del Serchio, Azienda Usl Toscana Nord Ovest, Via IV Novembre 10, 55027 Gallicano, Italy

**Keywords:** house fly (*Musca domestica*), *Staphylococcus* spp., coagulase-negative staphylococci, antimicrobial resistance

## Abstract

House flies (*Musca domestica*) are very diffuse insects attracted by biological materials. They are abundantly present in farm environments and can frequently come in contact with animals, feed, manure, waste, surfaces, and fomites; consequently, these insects could be contaminated, carry, and disperse several microorganisms. The aim of this work was to evaluate the presence of antimicrobial-resistant staphylococci in house flies collected in poultry and swine farms. Thirty-five traps were placed in twenty-two farms; from each trap, 3 different kinds of samples were tested: attractant material present in the traps, the body surface of house flies and the body content of house flies. Staphylococci were detected in 72.72% of farms, 65.71% of traps and 43.81% of samples. Only coagulase-negative staphylococci (CoNS) were isolated, and 49 isolates were subjected to an antimicrobial susceptibility test. Most of the isolates were resistant to amikacin (65.31%), ampicillin (46.94%), rifampicin (44.90%), tetracycline (40.82%) and cefoxitin (40.82%). Minimum Inhibitory concentration assay allowed to confirm 11/49 (22.45%) staphylococci as methicillin-resistant; 4 of them (36.36%) carried the *mecA* gene. Furthermore, 53.06% of the isolates were classified as multidrug-resistant (MDR). Higher levels of resistance and multidrug resistance were detected in CoNS isolated from flies collected in poultry farms than in swine farms. Therefore, house flies could carry MDR and methicillin-resistant staphylococci, representing a possible source of infection for animals and humans.

## 1. Introduction

Members of the *Staphylococcus* genus are ubiquitous bacteria. More than 80 species and subspecies exist, generally distinguished in Coagulase Positive Staphylococci (CoPS) and Coagulase Negative Staphylococci (CoNS) [1,2]. CoPS, mainly represented by *Staphylococcus aureus* subs. *aureus* (*S. aureus*) are important pathogens of humans and animals [2]. CoNS are opportunistic pathogens. However, in recent years, their virulence and pathogenic potential have been widely revaluated [3,4].

Like other bacteria, staphylococci could acquire resistance to several antimicrobials, representing a serious complication of staphylococcal infections [5,6]. One of the main problems related to *Staphylococcus* spp. is methicillin resistance. This characteristic is associated with the acquisition of a set of genes called staphylococcal cassette chromosome *mec* (SCC*mec*). These genes confer resistance to β-lactam antibiotics, including penicillins and cephalosporins [7,8]. The SCC*mec* harbors a gene called *mec* that encodes for a modified specific penicillin-binding protein (PBP2a) with a decreased binding affinity to β-lactams [7]. The *mecA* gene is the first identified and the most dispersed worldwide, but some homologous genes, such as *mecB* and *mecC*, exist [9]. The SCC*mec*, particularly harboring *mecA*, is mainly associated with *S. aureus*, and *S. aureus* strains carrying *mecA* are defined as methicillin-resistant *Staphylococcus aureus* (MRSA) [7]. Methicillin-resistant coagulase-negative staphylococci (MRCoNS) have been detected, too, and emerged as important opportunistic pathogens in recent years [10,11].

Both CoPS and CoNS are common inhabitants of skin and mucous membranes of domestic and wild animals, as well as humans, and can be considered typical zoonotic bacteria [1,2,12]. In addition, among domestic animals, swine and poultry are frequently carriers and reservoirs of staphylococci [13,14,15,16] and, potentially, the source of human infections [17,18].

In farm environments, insects could contribute to the spreading and persistence of pathogenic and antimicrobial-resistant bacteria. In particular, the role of flies was explored from this point of view [19,20,21]. However, most studies are focused on Gram-negative bacteria, particularly Enterobacteriaceae, whereas few data on flies as carriers of staphylococci at the farm level are available. Poudel and coworkers did not detect *S. aureus* in house flies sampled in poultry, dairy cattle, and beef cow farms. Still, they found CoNS in 85.00%, 67.50% and 82.76% of samples, respectively, 52.94%, 66.66% and 12.50% of isolates from poultry, dairy cattle, and beef cow, respectively, were resistant to one or more antimicrobials, too [22]. Akter and colleagues reported the isolation of *S. aureus* from 60% and 75% of *Musca domestica* samples collected in poultry and dairy farms, respectively [23]. Stelder and collaborators detected 7.8% and 5.4% of methicillin-resistant *S. aureus* strains in stable flies and house flies collected on a pig farm [24].

This study aimed to evaluate the presence of *Staphylococcus* spp. in *Musca domestica* collected in medium size poultry and swine farms located in central Italy, to assess the antimicrobial resistance of the isolates, and to evaluate the presence of methicillin-resistant staphylococcal strains.

## 2. Results

### 2.1. Farms and Samples

Seven poultry farms and fifteen swine farms were enrolled in the study. Ten traps were placed in poultry farms, while 25 were placed in swine farms. Overall, 105 samples were processed for microbiological investigation: 35 samples for each type A, B and C; sample A represent the attractant substrate inside the traps, sample B represents the insect’s external surface body and sample C the internal body content of the flies. Insects were captured with all the traps, and house flies alone were found in them. Detailed information about farms, traps and samples is resumed in Table 1.

### 2.2. Staphylococcus *spp*. Isolation, Characterization and Antimicrobial Resistance

*Staphylococci* were isolated from 23/35 (65.71%) traps from 16/22 (72.72%) farms. In detail, *Staphylococcus* spp. was detected in 16/35 (45.71%), 16/35 (45.71%) and 14/35 (40.00%) type A, B and C samples, respectively; no statistical differences emerged (*p* > 0.05). In some cases, the detection of these bacteria inside or on the body of insects (samples B and C) was not associated with the positivity of the broth inside the trap (sample A) and *vice versa*. Particularly, staphylococci were isolated from all 3 kinds of samples only in 8 traps. In 10 farms, more than 1 trap was placed; *Staphylococcus* spp. was isolated from all the traps within the same farm only in five cases. However, staphylococci were found in 9/10 (90.00%) farms where more than 1 trap was used and in 7/12 (58.33%) farms where only 1 trap was placed. Although using more traps allowed a more abundant detection of staphylococci, no statistical difference emerged (*p* > 0.05).

Overall, 46/105 (43.81%) samples resulted positive and 49 different *Staphylococcus* spp. isolates were collected and subsequently analyzed. Particularly, 18, 16 and 15 isolates were from type A, B and C samples, respectively. Only coagulase-negative staphylococci were found. In detail, the following species were identified: *S. epidermidis* (14 isolates), *S. xylosus* (9 isolates), *S. lentus* (7 isolates), *S. haemolyticus* (5 isolates), *S. sciuri* (5 isolates), *S. cohnii spp urealyticus* (4 isolates), *S. saprophyticus* (3 isolates) and *S. warneri* (2 isolates).

Considering swine, 10/15 (66.66%) farms, 15/25 (60.00%) traps, and 28/75 (37.33%) samples were positive. Whereas, considering poultry, 6/7 (85.71%) farms, 8/10 (80.00%) traps and 18/30 (60.00%) samples were positive. No statistical differences emerged in the positivity rate between swine and poultry farms and traps (*p* > 0.05). In contrast, more samples collected from poultry were positive than samples collected from swine settings (*p* < 0.05).

Table 2 summarizes data about antimicrobial resistance in *Staphylococcus* spp. isolates.

High percentages of resistance were detected for amikacin (65.31% resistant isolates), ampicillin (46.94% resistant isolates), rifampicin (44.90% resistant isolates), tetracycline (40.82% resistant isolates) and cefoxitin (40.82% resistant isolates). The most effective antimicrobial resulted in amoxicillin-clavulanate (85.71% susceptible isolates), chloramphenicol (75.51% susceptible isolates), trimethoprim-sulfamethoxazole (71.43% susceptible isolates), ciprofloxacin (69.39% susceptible isolates), gentamicin (69.39% susceptible isolates) and ceftiofur (65.31% susceptible isolates).

None of the isolates was resistant to vancomycin. In particular, 40/49 (81.63%) isolates were susceptible, and the remaining 9/49 (18.37%) were classified as intermediate.

The 20 isolates resistant to cefoxitin were tested to determine oxacillin MIC. Among them, 11/20 (55.00%) were confirmed as methicillin-resistant, and 9/20 (45.00%) resulted susceptible. In addition, four out of eleven phenotypic-resistant strains scored positive for the gene *mecA*; the gene *mecC* was undetected.

For the following antimicrobials, a higher percentage of resistant staphylococci was detected among isolates from poultry than among isolates from swine: cefoxitin, ceftiofur, enrofloxacin, gentamicin, amikacin, erythromycin, and trimethoprim-sulfamethoxazole (*p* < 0.05).

No differences emerged between isolates from swine and poultry in relation to vancomycin resistance and methicillin resistance (*p* > 0.05). Furthermore, 2 *mecA-positive* strains were recovered from swine and 2 from poultry samples.

*Staphylococcus* spp. isolates were resistant from 0 to 10 different antimicrobials. Based on the antimicrobial resistance profile, 26/49 (53.06%) isolates were classified as multidrug-resistant (MDR), showing resistance to at least 1 antimicrobial in three or more different antimicrobial classes [25]. It was possible to isolate MDR strains from 14/22 (63.64%) farms. In particular, 6/7 (85.71%) and 8/15 (53.33%) poultry and swine farms were positive for MDR strains, respectively; no statistical differences emerged (*p* > 0.05). It was possible to isolate MDR strains from 16/35 (45.71%) traps. In particular, from 7/10 (70.00%) and 9/25 (36.00%), traps placed in poultry and swine farms, MDR strains were detected, respectively; no statistical differences emerged (*p* > 0.05). Finally, 25/105 (23.81%) samples were positive for MDR staphylococci: 8/35 (22.86%), 9/35 (25.71%) and 8/35 (22.86%) type A, B and C samples, respectively; no statistical differences were detected (*p* > 0.05). In particular, 14/30 (46.67%) and 11/75 (14.67%) samples collected from poultry and swine farms allowed the isolation of MDR strains; samples from poultry farms allowed more often the isolation of MDR staphylococci (*p* < 0.05). 

All methicillin-resistant isolates were multidrug-resistant.

Table 3 reports detailed data on MDR isolates obtained in this study.

## 3. Discussion

The present study has investigated the potential role of house flies in the carriage and dispersion of antimicrobial-resistant staphylococci in medium-size swine and poultry farms.

Three different kinds of samples were evaluated. Sample A, the attractant broth present inside the trap, gives information about environmental contamination and the contamination carried out by insects. Sample B, the rinsing solution of the external body of the flies, provides information on the staphylococci present on the surface of the captured house flies. Finally, sample C, the homogenate of the insect bodies, provides data about the staphylococci inside the flies. A more detailed evaluation of the importance and meaning of the evaluation of all three samples was previously published [26].

In the present study, *Staphylococcus* spp. were isolated from more than 60% of traps and in more than 70% of farms, confirming the high diffusion of bacteria belonging to this genus. However, less than 50% of samples scored positive, without differences among samples A, B and C; furthermore, staphylococci were isolated from all three kinds of samples only in a little number of traps (8/35—22.86%). Acquired data suggested that the external surface and the digestive tract of house flies could be contaminated by *Staphylococcus* spp. Only CoNS were detected; this result is unsurprising because CoNS are more frequently detected than CoPS in healthy swine or poultry [13,14,15]. Obtained data correspond to other studies where CoNS were more frequently detected in house flies than CoPS [22]. Sobur and colleagues reported a high isolation rate (52%) of *S. aureus* in house flies. However, in this case, sampling was performed inside or near a human hospital, and this could explain the frequent detection of this pathogen [27].

The most effective antimicrobials were amoxicillin-clavulanate, chloramphenicol, trimethoprim-sulfamethoxazole, ciprofloxacin, gentamicin and ceftiofur, with more than 60% of isolates resulting susceptible. More than 40% of tested staphylococci were resistant to amikacin, ampicillin, rifampicin, tetracycline and cefoxitin. In 2019 and 2020, in Italy, the most sold antimicrobials for farm animals were penicillins and tetracyclines, followed by sulfonamides, lincosamides and aminoglycosides [28]; this could partially explain the resistance detected. However, a real comparison with other works is difficult to perform. First, few studies exploring the presence and antimicrobial resistance of staphylococci in house flies are available. Second, because the panels of antimicrobials tested differ among the studies, and third, because detected antimicrobial resistance reflects the location and geographic area where insects come from. In a survey about bacteria from flies captured in hospital and non-hospital settings, CoPS and CoNS showed moderate resistance to ciprofloxacin only [29]. A study performed on house flies captured in different locations, including animal facilities, reported an increased resistance only for tetracyclines and amoxicillin, whereas most of the tested staphylococcal isolates were susceptible to the other tested antimicrobials [22]. Akter et al. evaluated the antimicrobial resistance of *S. aureus* strains isolated from house flies collected in different settings, including poultry and dairy farms; most strains were resistant to amoxicillin, penicillin, streptomycin, erythromycin, and tetracycline, whereas most effective antimicrobials were ciprofloxacin and chloramphenicol [23]. Odetoyin and collaborators analyzed *S. aureus* strains from house flies collected in different locations. The antimicrobial susceptibility test showed diffuse resistance to amoxicillin and trimethoprim-sulfamethoxazole and susceptibility to chloramphenicol, amoxicillin-clavulanate, gentamycin and ciprofloxacin [30]. Sobur and coworkers found a high percentage of *S. aureus* resistant to amoxicillin and oxacillin in house flies collected near a human hospital; most strains were susceptible to ciprofloxacin and gentamicin [27]. In a recent study on CoNS isolated from house flies collected from different environments, a high percentage of tested staphylococci were resistant to oxacillin and penicillin but susceptible to tetracycline, ciprofloxacin, gentamicin, and chloramphenicol [31].

One of the main problems associated with *Staphylococcus* spp. is methicillin resistance; it is related to a mobile genetic element carrying *mecA* or homologous genes, conferring broad-spectrum β-lactam resistance [7]. In the present study, 22.44% of isolated staphylococci were methicillin-resistant by phenotypic test. Only 4 isolates (36.36% of methicillin-resistant and 8.16% of the total tested isolates) carried the *mecA* gene, whereas *mecC* was undetected. Sobur and collaborators found higher percentages of oxacillin resistant (84.13%) and *mecA-positive* (57.7%) staphylococci collected from house flies; however, these authors focused on *S. aureus* and flies collected in a human hospital setting [27]. Akter and collaborators reported a higher percentage of *mecA-positive* staphylococci in house flies. In this case, only *S. aureus* was tested, and most MRSA was isolated from insects collected in a human hospital [23]. Other authors reported a high percentage (90.63%) of oxacillin-resistant CoNS isolated from house flies but the absence of *mecA-positive* strains [31]. Molecular detection of *mecA* or *mecC* genes was considered the gold standard method and the key point to classify a *Staphylococcus* strain as methicillin-resistant. However, other *mec* homologous exist; these genes are less diffuse and generally not located on mobile genetic elements [7], and for this were not searched in our study. Furthermore, other resistance mechanisms were identified, especially in *S. aureus*, such as overexpression of β-lactamases producing genes, for example, *blaZ*, or point mutation of PBP [32,33,34].

Vancomycin was used in humans to treat methicillin-resistant *Staphylococcus* infections [35]. All tested isolates obtained in the present study were classified as susceptible or intermediate against vancomycin. This aligns with other studies, reporting no detection of vancomycin-resistant CoNS [36,37].

In the present survey, 53.06% of isolates were multidrug resistant; it was possible to detect MDR strains from more than 50% of swine and almost all poultry farms. The resistance to multiple antimicrobials represents an excellent advantage for bacteria and a serious threat to humans and veterinary medicine, limiting the available therapeutic options. Our data contrast with other studies using the same criteria to classify multidrug-resistant staphylococci. Indeed, lower percentages of MDR CoNS were reported by other authors, ranging between 0.8% and 12.5% [22,31]. However, the MDR detection rate from house flies similar to our study was obtained by some authors analyzing *S. aureus* strains and adopting the same MDR classification criteria [27,30].

Although no differences emerged in the detection rate of staphylococci between house flies collected in swine and poultry farms, antimicrobial resistance was higher in isolates of poultry origin. Indeed, for 7 out of 14 antimicrobials tested, a higher percentage of resistant isolates was detected in staphylococci from poultry farms than from swine farms. Furthermore, MDR strains were more often detected from samples collected in poultry farms. Official data report a similar quantity of sold antimicrobials in Italy for swine and poultry in recent years [28]. However, in this study, small-medium size farms were analyzed, and we could hypothesize a stronger use of antimicrobials in poultry flocks. In addition, results of other works seem to suggest a more abundant use of antimicrobials in poultry farms, reporting higher detection of antimicrobial-resistant bacteria or antimicrobial-resistance genes [38,39,40].

## 4. Materials and Methods

### 4.1. Farms, Traps, Insects Collection and Processing

Samples collection and processing methods were previously described [26]. Briefly, samples were collected from June 2019 to September 2019 in poultry and swine farms located in North-west Tuscany, Central Italy, under the area of competence of “Az. USL di Versilia, Valle del Serchio e Piana di Lucca”.

Home-made traps were used to capture the insects; sterile glass jars filled with freshly prepared and sterilized fish broth as attractant substrate were employed. Traps were located inside the animal breeding rooms, far from windows and doors and not accessible to animals.

Three different samples (A, B, C) were obtained from each trap. First, the attractant material remaining inside the trap was collected and analyzed. This represents sample A. The house flies external surface body was washed with a sterile saline solution, and the washing solution was used as sample B. Finally, the insect bodies were decontaminated and homogenized in a sterile saline solution with a Stomacher. The obtained homogenate represented sample C.

### 4.2. Staphylococcus *spp.* Isolation

To isolate bacteria from the *Staphylococcus* genus, 1 mL of each sample (A, B and C) was diluted from 10^−1^ to 10^−3^ in sterile saline water. Successively, 0.1 mL from each dilution and the original sample were inoculated with the spread-plate technique on Mannitol Salt Agar (MSA) (Thermo Fisher Diagnostics, Milan, Italy) to obtain single isolated colonies; plates were incubated at 37 °C for 24 h. From each sample, up to 3 distinct and different colonies were selected and purified on Tryptic Soy Agar (TSA) (Thermo Fisher Diagnostics). Isolates were confirmed by Gram staining and catalase tests. *Staphylococcus* spp. isolates were further tested for coagulase with rabbit plasma (Biolife, Milan, Italy); 1 to 3 isolates from each sample were selected based on the coagulase test and mannitol fermentation. Species identification was carried out with API STAPH^®^ (bioMérieux SA, Marcy l’Etoile, France) following the manufacturers’ instructions. All typed isolates were cultured in Brain Heart Infusion broth (BHI) (Thermo Fisher Diagnostics) and frozen at −80 °C with the addition of 20% glycerol.

### 4.3. Antimicrobial Susceptibility Tests

All obtained isolates were tested for antimicrobial resistance with the disk diffusion method described by CLSI [41]. The following antimicrobial (Thermo Fisher Diagnostics) were employed: ampicillin (10 µg), amoxicillin-clavulanate (20/10 μg), cefoxitin (30 μg), ceftiofur (30 μg), chloramphenicol (30 μg), tetracycline (30 μg), enrofloxacin (5 μg), ciprofloxacin (5 μg), gentamicin (10 μg), amikacin (30 μg), trimethoprim-sulfamethoxazole (1.25/23.75 μg), erythromycin (15 μg) and rifampicin (5 μg). Vancomycin resistance of staphylococci isolates was evaluated, too; as suggested by CLSI, Minimum Inhibitory Concentration (MIC) was assessed for this purpose, using the broth microdilution method [42]. Results were interpreted in accordance with CLSI and EUCAST guidelines [42,43,44]. *Escherichia coli* ATCC 25922, *Staphylococcus aureus* ATCC 25923 and *Staphylococcus aureus* ATCC 29213 were internal quality controls.

Oxacillin MIC was evaluated for *Staphylococcus* spp. isolates resulting resistant or intermediate to cefoxitin [42]. Isolates confirmed as resistant were subjected to molecular detection of *mecA* and *mecC* genes employing primers (Table 4), and PCR protocols previously described [45,46]. DNA was extracted from overnight cultures using a commercial kit, Quick-DNA Miniprep Plus Kit (Zymo Research, Irvine, CA, USA), following manufacturer instructions. PCR assays were done in an automated thermal cycler (SimpliAmp™ Thermal Cycler, Applied Biosystems, Waltham, MA, USA). PCRs reactions were carried out in 25 µL final volume, containing 12.5 µL DreamTaq Hot Start Green Master Mix (Life Technologies Italia, Milan, Italy), 0.1 µM of primers MecA147-F and MecA147-R, or 0.5 μM of primers mecLGA251 f and mecLGA251 r, 3 µL of extracted DNA and ultrapure water to reach the final volume. Sterile distilled water was employed as negative control; DNA extracted from *Staphylococcus aureus* ATCC 43300 was used as a positive control for *mecA.* The DNA extracted from previously isolated and characterized field strains was used as a positive control for *mecC*. PCR products were run in 1.5% agarose gel at 100 V for 45 min, using 100 bp DNA Ladder Ready to Load (Solis BioDyne, Tartu, Estonia) as DNA marker; the gel was stained with ethidium bromide and observed under UV light.

### 4.4. Statistical Analyses

Obtained results were analyzed with Chi-square (X^2^) test. The Chi-square (X^2^) test was employed to compare isolation rates and antimicrobial resistance of staphylococci between poultry and swine farms and among sample types. The statistical significance threshold was set at a *p*-value ≤ 0.05.

## 5. Conclusions

Data obtained in the present investigation confirm that house flies could carry antimicrobial-resistant staphylococci representing a potential vector for bacterial dispersion. Only CoNS were isolated from the analyzed samples; these bacteria act as opportunistic pathogens and are frequently involved in human and animal infections. A high proportion of isolated bacteria were multidrug-resistant, suggesting an abundant circulation of MDR staphylococci in farmed animals. Although a low proportion of methicillin-resistant and *mecA-positive* CoNS was detected, our data show the circulation of these bacteria in medium-small poultry and swine farms. They suggest that *M. domestica* could be relevant in spreading MDR and methicillin-resistant coagulase-negative staphylococci.

House flies are common insects in every ecosystem and abundantly diffuse in farm environments. As a result, they can easily and frequently come in contact with animals’ bodies, representing an irritant and, sometimes, stressful factor. A good biosecurity plan to reduce the number of houses flies in farm environments could help reduce animals’ stress and prevent the dispersion of antimicrobial-resistant, potentially pathogenic bacteria.

## Figures and Tables

**Table 1:** Positive farms, traps, and samples to *Staphylococcus* spp.

Farms	Farmed Animals	Traps ID	Number of Positive Traps to *Staphylococcus* spp.	Positive Samples to *Staphylococcus* spp.		
				A	B	C
1	Poultry	1	2/2	+	+	+
		2		+	−	−
2	Swine	3	0/1	−	−	−
3	Swine	4	1/1	+	+	−
4	Poultry	5	1/1	+	+	+
5	Poultry	6	1/2	−	−	−
		7		+	+	+
6	Poultry	8	0/1	−	−	−
7	Poultry	9	1/1	+	−	−
8	Swine	10	2/2	+	+	−
		11		+	−	+
9	Swine	12	1/1	−	+	−
10	Swine	13	1/1	−	−	+
11	Poultry	14	2/2	+	+	+
		15		−	−	+
12	Swine	16	0/1	−	−	−
13	Swine	17	1/2	−	−	−
		18		+	+	−
14	Swine	19	0/1	−	−	−
15	Swine	20	1/1	+	+	+
16	Swine	21	0/1	−	−	−
17	Poultry	22	1/1	+	+	+
18	Swine	23	3/3	−	−	+
		24		+	+	−
		25		−	+	−
19	Swine	26	0/2	−	−	−
		27		−	−	−
20	Swine	28	2/2	−	+	+
		29		+	+	−
21	Swine	30	2/3	−	−	+
		31		−	−	−
		32		+	+	+
22	Swine	33	1/3	−	−	−
		34		−	−	−
		35		+	+	+

**Table 2:** Results of disk diffusion test carried out on *Staphylococcus* spp. isolates.

Antimicrobial	Susceptible		Intermediate		Resistant	
	N° of Isolates	%	N° of Isolates	%	N° of Isolates	%
Ampicillin	26	53.06	0	0.00	23	46.94
Amoxicillin-clavulanate	42	85.71	0	0.00	7	14.29
Cefoxitin	29	59.18	0	0.00	20	40.82
Ceftiofur	32	65.31	4	8.16	13	26.53
Chloramphenicol	37	75.51	4	8.16	8	16.33
Tetracycline	25	51.02	4	8.16	20	40.82
Enrofloxacin	25	51.02	16	32.65	8	16.33
Ciprofloxacin	34	69.39	8	16.33	7	14.29
Gentamicin	34	69.39	2	4.08	13	26.53
Amikacin	17	34.69	0	0.00	32	65.31
Trimethoprim-sulfamethoxazole	35	71.43	3	6.12	11	22.45
Erythromycin	8	16.33	22	44.90	19	38.78
Rifampicin	21	42.86	6	12.24	22	44.90

**Table 3:** Antimicrobial resistance profile of the multidrug-resistant *Staphylococcus* spp. isolates.

Isolate Number	Farm	Farmed Animal	Traps ID	Sample Type	Species	Antimicrobial Resistance Profile
01Aa	1	Poultry	1	A	*S. xylosus*	AMP AMC FOX EFT TE ENR CIP CN AK E
01Ab	1	Poultry	1	A	*S. lentus*	AMP AMC EFT TE ENR AK SXT E RD
01B	1	Poultry	1	B	*S. xylosus*	AMP EFT TE ENR CIP CN AK E RD
01C	1	Poultry	1	C	*S. epidermidis*	AMP FOX EFT ENR CIP CN AK RD
02A	1	Poultry	2	A	*S. warneri*	AMP AMC FOX EFT TE CN AK SXT E RD
04A	3	Swine	4	A	*S. epidermidis*	AMP AMC FOX EFT C CN AK E RD *
04B	3	Swine	4	B	*S. epidermidis*	AMP FOX EFT C TE CIP CN AK E RD *
05A	4	Poultry	5	A	*S. lentus*	EFT TE AK SXT E RD
05B	4	Poultry	5	B	*S. saprophyticus*	AMP FOX C TE CN AK SXT E RD
05C	4	Poultry	5	C	*S. haemolyticus*	AMP EFT TE ENR CN AK RD
07A	5	Poultry	7	A	*S. epidermidis*	AMP AMC FOX EFT C TE ENR CIP CN AK *
07B	5	Poultry	7	B	*S. epidermidis*	AMP AMC AK SXT E RD
07C	5	Poultry	7	C	*S. epidermidis*	AMP FOX C TE ENR CIP CN AK SXT E *
09A	7	Poultry	9	A	*S. lentus*	TE CN AK E RD
10Ab	8	Swine	10	A	*S. xylosus*	C TE E
10B	8	Swine	10	B	*S. epidermidis*	AMP ENR CIP CN AK SXT RD
12B	9	Swine	12	B	*S. xylosus*	AMP EFT TE E RD
13C	10	Swine	13	C	*S. chonii* ssp. *urealyticus*	AMP FOX EFT TE CN AK SXT ERD
14A	11	Poultry	14	A	*S. chonii* ssp. *urealyticus*	AMP AK E
18B	13	Swine	18	B	*S. sciuri*	AMP AK RD
22B	17	Poultry	22	B	*S. haemolyticus*	AK SXT RD
22C	17	Poultry	22	C	*S. haemolyticus*	FOX TE AK SXT RD
23C	18	Swine	23	C	*S. xylosus*	AMP AMC C TE E RD
24B	18	Swine	24	B	*S. xylosus*	AMP E RD
28C	20	Swine	28	C	*S. epidermidis*	AMP FOX EFT AK RD
30C	21	Swine	30	C	*S. xylosus*	AMP AK E

Legend: AMP = Ampicillin, AMC = Amoxicillin-clavulanate, FOX = Cefoxitin, EFT = Ceftiofur, C = Chloramphenicol, TE = Tetracycline, ENR = Enrofloxacin, CIP = Ciprofloxacin, CN = Gentamicin, AK = Amikacin, SXT = Trimethoprim-sulfamethoxazole, E = Erythromycin, RD = rifampicin; * = *mecA* positive.

**Table 4:** Primers employed in the study and related relevant information.

Gene	Primer	Oligonucleotide Sequence (5′-3′)	Annealing Temperature	Amplicon Size (bp)	Reference
*mecA*	MecA147-F	GTGAAGATATACCAAGTGATT	50 °C	147	[46]
	MecA147-R	ATGCGCTATAGATTGAAAGGAT			
*mecC*	mecLGA251 f	GCTCCTAATGCTAATGCA	50 °C	304	[45]
	mecLGA251 r	TAAGCAATAATGACTACC			

## Data Availability

The data presented in this study are available within the article.
